# Successful recanalization for internal carotid artery occlusion with persistent primitive trigeminal artery manifesting only as ischemia of the posterior circulation

**DOI:** 10.1186/s12883-016-0559-6

**Published:** 2016-03-22

**Authors:** Ryo Hiramatsu, Hiroyuki Ohnishi, Shinji Kawabata, Shigeru Miyachi, Toshihiko Kuroiwa

**Affiliations:** Department of Neurosurgery, Osaka Medical College, 2-7 Daigaku-machi, Takatsuki City, Osaka 569-8686 Japan

**Keywords:** Persistent primitive trigeminal artery, Internal carotid artery occlusion, Basilar artery occlusion, The ischemia of the posterior circulation, Penumbra system

## Abstract

**Background:**

Internal carotid artery (ICA) occlusion mainly manifests as ischemia of the anterior circulation. There are very few reports of ICA occlusion manifesting as only ischemia of the posterior circulation related to a fetal type posterior communicating artery or other arteries.

**Case presentation:**

The authors experienced a case of ICA occlusion with persistent primitive trigeminal artery (PPTA) manifesting only as ischemia of the posterior circulation. In this case, the initial NIHSS score was high (35/42 points). Additionally, cross flow of the anterior communicating artery, ICA occlusion and basilar artery (BA) occlusion were represented on the initial head MRA. Therefore, our first impression was a presumptive diagnosis of BA occlusion. Prior head MRI/MRA performed for screening purposes, had incidentally demonstrated a right PPTA. Based on this understanding, we were able to determine the exact angioarchitectural mechanism of the ICA occlusion. Because of the presence of the PPTA, successful recanalization was accomplished expeditiously.

**Conclusion:**

Although the presence of PPTA is rare and ICA occlusion patients with PPTA is even more unusual, if ICA occlusion and BA occlusion appear simultaneously on MRA, the presence of PPTA should be considered.

## Background

Acute internal carotid artery (ICA) occlusions are often associated with poor outcomes and severe neurologic deficits [1, 2]. Acute ICA occlusions are more resistant than MCA occlusions to administration of intravenous recombinant tissue-type plasminogen activator (rt-PA) [3, 4]. In the Trial of Org 10172 in Acute Stroke Treatment, 10 % of patients were diagnosed with ICA occlusion, which resulted in neurologic disability in 40 % and mortality in 20 % of patients [5]. ICA occlusion mainly manifests as ischemia of the anterior circulation. There are very few reports of ICA occlusion manifesting only as ischemia of the posterior circulation related to the fetal type posterior communicating artery or any other arteries [6]. We experienced a case of ICA occlusion with persistent primitive trigeminal artery (PPTA) manifesting only as ischemia of the posterior circulation.

PPTA is the most common anomaly of carotid-vertebrobasilar anastomoses occurring during embryological development of the intracranial vasculature. The incidence of PPTA has been reported to be between 0.1 and 0.6 % on the basis of conventional angiography and MRA findings [7–9]. Although 25 % of PPTA are associated with intracranial vascular anomalies such as aneurysms (13.8 %) [10] and arteriovenous malformations (4.5 %) [11], little is known about the significance of a PPTA in occlusive cerebrovascular disease.

## Case presentation

**Fig. 1:**
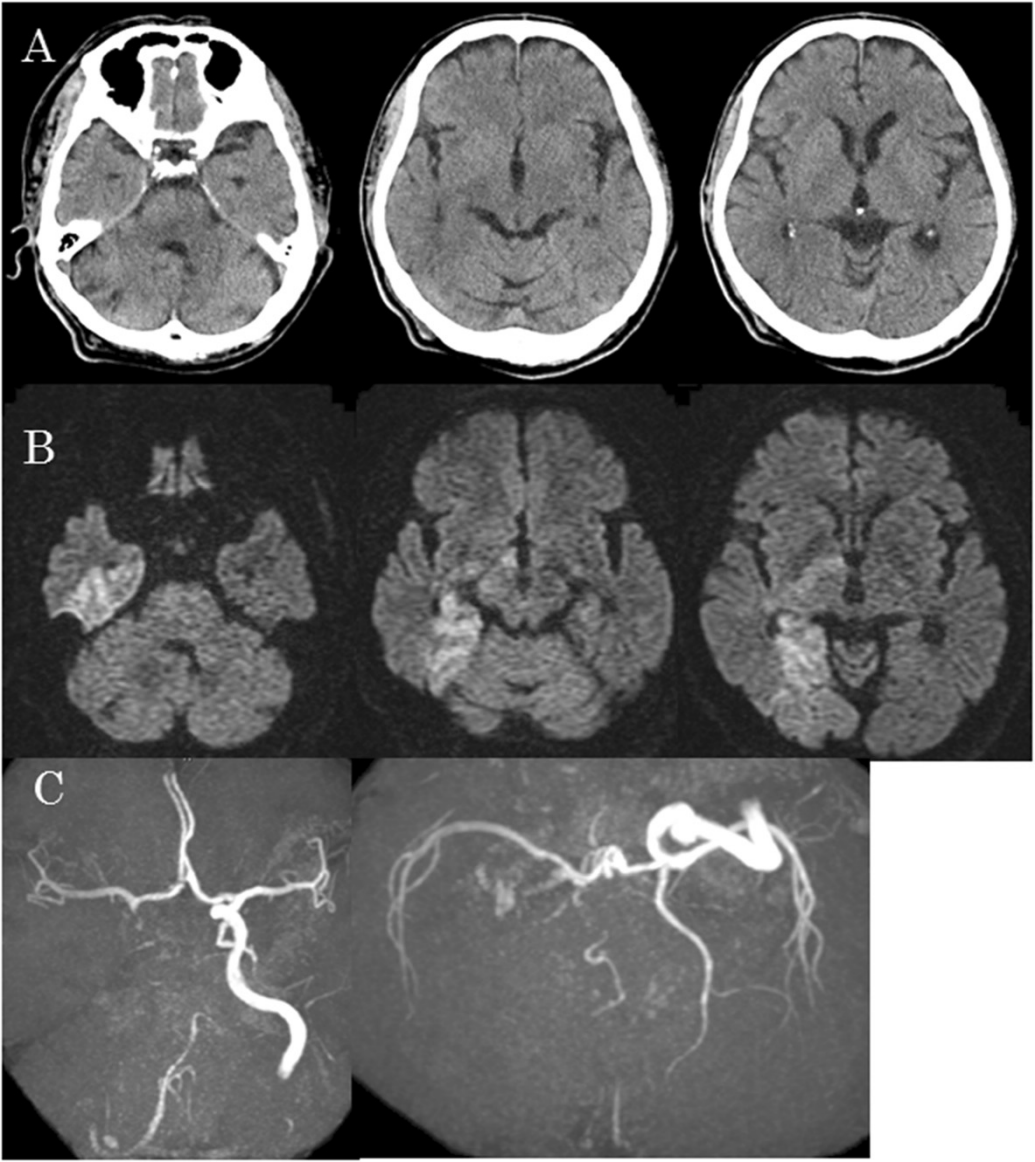
Preoperative head CT and head MRI/MRA. **a** Initial head CT showed no early ischemic signs. **b** The onset head MRI DWI showed HIA in the right medial temporal lobe, right hypothalamus, right internal capsule and right medial occipital lobe. **c** Initial head MRA showed right ICA occlusion and BA occlusion. However, right anterior and middle cerebral artery were represented clearly by virtue of the Acom

**Fig. 2:**
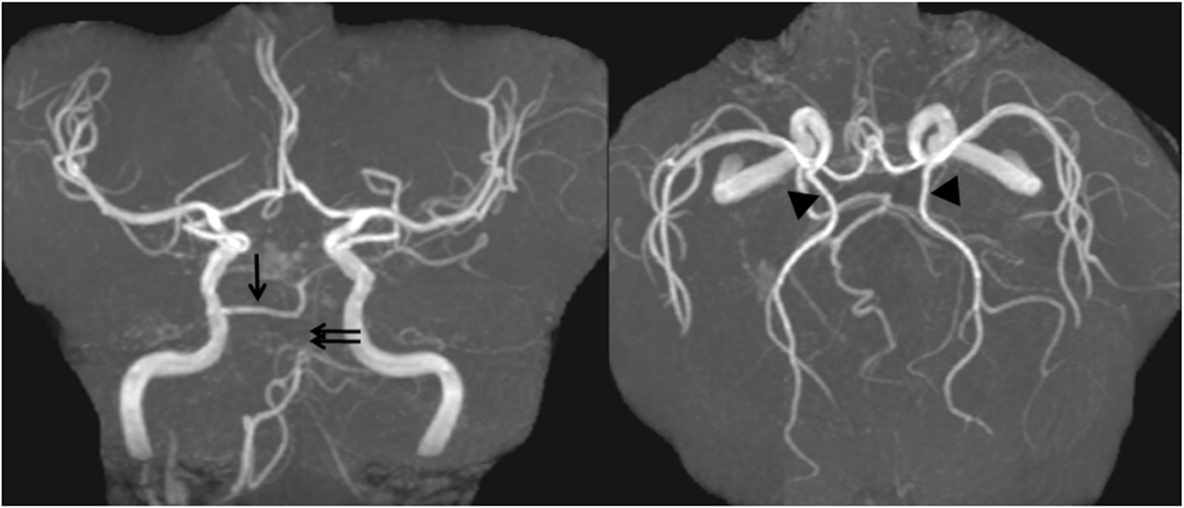
Prior head MRA. Prior radiological MRA studies show a right PPA (*single black arrow*). This PPTA joins the middle basilar artery proximal to SCA and leads to the end of the SCA (*double black arrows*). Bilateral PCAs received their blood supply predominantly through the patent PCom (*black arrow heads*)

A 65-year-old Japanese man with a history of atrial fibrillation (treated with oral warfarin) and hypertrophic cardiomyopathy suffered sudden onset of stertorous breathing, followed by deteriorated into coma in December of 2014. The patient was transported to our hospital by ambulance, and on admission he was experiencing ataxic breathing. Initial National Institute of Health Stroke Scale (NIHSS) was 35/42 points with a Glasgow Coma Scale of 7 (E:1, V:2, M:4). Laboratory examination revealed a prothrombin time-international normalized ratio (PT-INR) of 1.87. There was no evidence of early ischemic signs on head CT (Fig. 1a). However a high intensity area (HIA) was visible in the right medial temporal lobe, right hypothalamus, right internal capsule and right medial occipital lobe on head MRI diffusion-weighted imaging (DWI) (Fig. 1b). Head MRA showed right ICA occlusion and basilar artery (BA) occlusion (Fig. 1c). However, right anterior and middle cerebral arteries were patent by virtue of flow via the anterior communication artery (Acom) (Fig. 1c). Prior head MRI/MRA performed for screening purposes, had incidentally demonstrated a right PPTA (Fig. 2). This PPTA joined the middle BA proximal to the superior cerebellar artery (SCA) and fed branched SCAs. Because the initial NIHSS score was high (35 points) and there was clear cross flow from the Acom, BA occlusion was suspected. However, PPTA was not visible in the initial MRA. Therefore, ICA occlusion with PPTA involvement postulated. Because PT-INR was 1.87, rt-PA intravenous therapy was not utilized and instead endovascular therapy was performed.

**Fig. 3:**
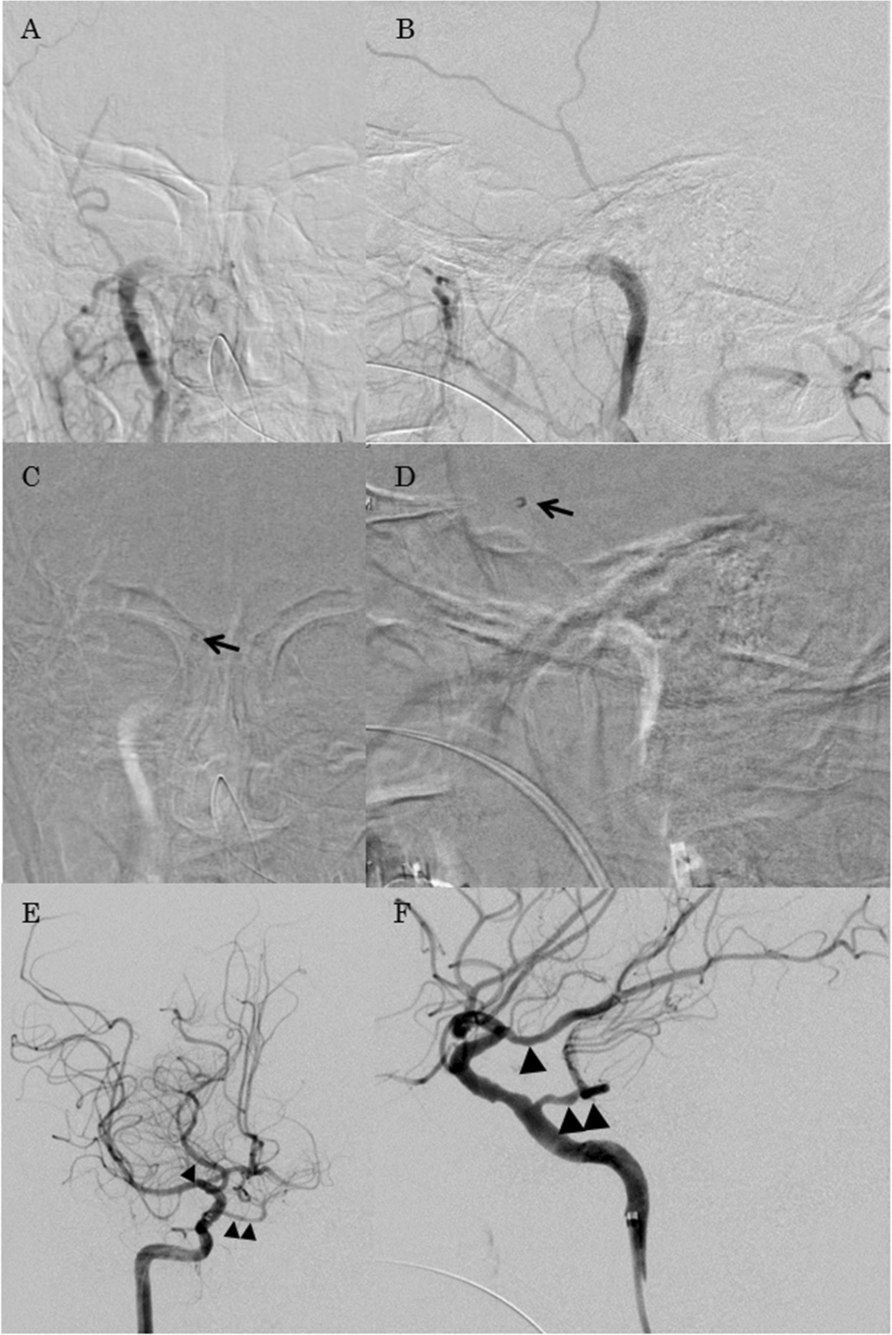
Operative photographs. **a** Right ICAO was shown on an anterior-posterior view of the preoperative angiography. **b** Right ICAO was shown on a lateral view of the preoperative angiography. **c** The 5MAX ACE Reperfusion Catheter (*single black arrow*) was guided to the thrombus proximal to the PPTA on an anterior-posterior view of the intraoperative angiography. **d** The 5MAX ACE Reperfusion Catheter (*single black arrow*) was guided to the thrombus proximal to the PPTA on a lateral view of the intraoperative angiography. **e** Successful recanalization achieved a TICI 3 score as shown on an anterior-posterior view of the postoperative angiography. Photographically, Pcom of a fetal type (*single black arrow head*) and PPTA (*double black arrow heads*) were shown. **f** Successful recanalization achieved a TICI 3 score as shown on a lateral view of the postoperative angiography. Photographically, Pcom of a fetal type (*single black arrow head*) and PPTA (*double black arrow heads*) were shown

**Fig. 4:**
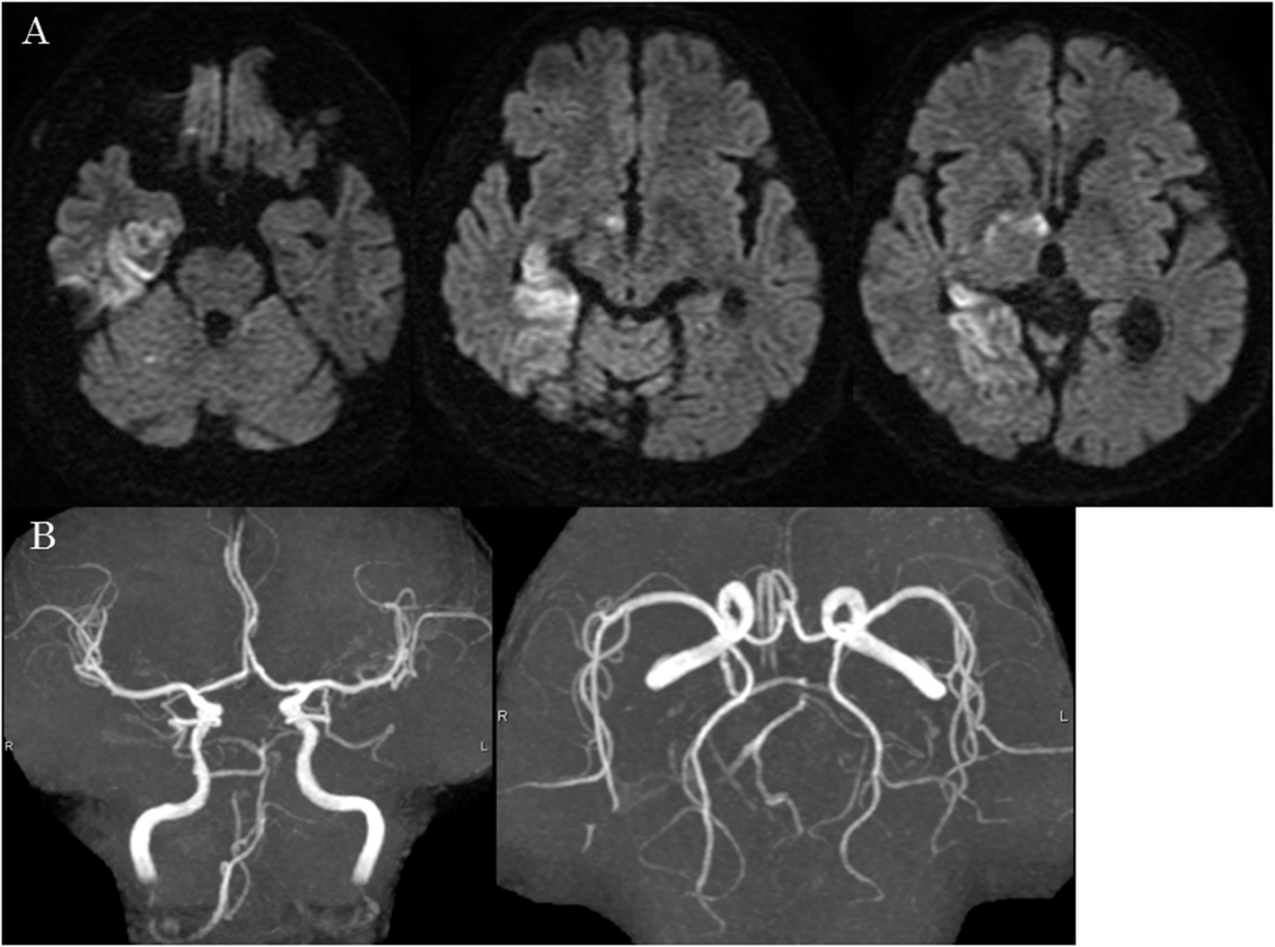
Postoperative MRI/MRA. **a** There were no new acute ischemic lesions on postoperative MRI DWI. **b** All cerebral vessels including PPTA were represented on the postoperative MRA

We inserted a 9Fr long sheath in the right femoral artery. 9Fr Optimo 90 STR (Tokai Medical Products) was placed in the right internal carotid artery at the C1 level. Digital subtraction angiography revealed right ICA occlusion (Fig. 3a). A coaxial system which consisted of 5MAX ACE Reperfusion Catheter (Penumbra, Inc.), 3MAX ACE Reperfusion Catheter (Penumbra, Inc.) and CHIKAI 14 (ASAHI INTECC CO.) was inserted in the Optimo. The 5MAX ACE was guided to the thrombus proximal to the right PPTA and was connected at this point to the Aspiration Pump (Penumbra, Inc.). We guided the 5MAX ACE distally to the right posterior communication artery (Pcom) and all blood clots were aspirated. After a first pass of the direct aspiration using the 5MAX ACE, we achieved successful recanalization with a Thrombolysis in Cerebral Infarction score (TICI) of 3 (Fig. 3b). Postoperatively there were no new acute ischemic lesions on postoperative MRI DWI (Fig. 4a), and all cerebral vessels including PPTA were visible on postoperative MRA (Fig. 4b). The patient was discharged on postoperative day 13, with a normal neurological examination except for the presence of an upper left quadrantanopia.

## Discussion

The PPTA is one of the four well known primitive anastomoses between the internal carotid artery and the vertebrobasilar system (the hypoglossal, otic, and proatlantal intersegmental arteries comprise the remaining three). It accounts for approximately 80–85 % of persistent anastomoses [12]. The PPTA originates from the cavernous segment of the ICA and communicates with the BA [13]. Saltzman et al. [14] classified the angiographic appearance of PPTA by conventional angiography into three types according to the angioarchitectural relationship with the neighboring vessels. Cases may be encountered that do not meet Saltzman et al.’s classification, making it difficult to understand the various types of PPTA. Recently, Weon et al. [15] proposed a new classification scheme for PPTA based on MRA, identifying five different types based on their anatomical relationship with the neighboring arteries. According to Weon et al. classification, the PPTA in our case was type 2 [15].

PPTA is frequently associated with other anatomical variations such as absence of the ipsilateral Pcom, vertebral artery, or BA [16, 17]. In a large case series, approximately 75 % of cases demonstrated different grades of BA hypoplasia [9]. In these circumstances, the BA received its flow almost exclusively from the ICA with supply to the upper brain stem, cerebellum and ipsilateral cerebral hemisphere arising from the ICA [16]. However patients with PPTA who developed ICA occlusion with cross flow from the Acom, are at high risk of severe cerebral infarction and associated BA top syndrome.

In our case, the initial NIHSS score was high (35 points) and Acom cross flow was represented on the initial head MRA. Therefore, our first impression was a presumptive diagnosis of BA occlusion. However, reference to prior head MRA, provided additional information necessary for a diagnosis of ICA occlusion, not BA occlusion. Additionally, HIA on the initial MRI DWI showed a lesion limited to the posterior cerebral artery (PCA) area. The PPTA in our case was Weon et al.’s classification type 2 (Fig. 2), which is defined as PCA blood supply predominantly from a patent PCom [15]. Based on this understanding, we were able to determine not only the exact angioarchitectural mechanism of the ICA occlusion but also the location of the thrombus (probably, it located at the origin of PPTA continuing to the origin of Pcom). Ultimately, we were able to attain a good result (a modified Ranking Scale of 1 at discharge). Another factor contributing to the good result might be the use of the Penumbra System, not a stent retriever. In this case, because sufficient cross flow from the Acom was observed on the initial head MRA, the thrombectomy might lead to incidental embolization in new territories (ENT) or embolization of distal territories (EDT) with accompanying Acom occlusion. Mokin et al. reported that an use of direct aspiration (using a 5MAX ACE Reperfusion Catheter) showed less ENT and EDT than the stent retriever in in vitro model experiments [18]. On the other hand, Mulder et al. reported the thrombectomy in posterior circulation stroke through persistent primitive trigeminal artery using a stent retriever as a case report [19]. Therefore, even though it is still controversial which devices (Penumbra System or stent retriever) can show less ENT and EDT, the Penumbra System was critically useful in our case.

## Conclusions

Although the presence of PPTA is rare and ICA occlusion in patients with PPTA is even more unusual, if ICA occlusion and BA occlusion appear simultaneously on MRA or other imaging, the presence of PPTA should be considered.

## Consent

Written informed consent was obtained from the patient for publication of this Case report and any accompanying images. A copy of the written consent is available for review by the Editor of this journal.

## Abbreviations

Acom: anterior communicating artery
BA: basilar artery
DWI: diffusion-weighted imaging
EDT: embolization of distal territories
ENT: embolization in new territories
HIA: high intensity area
ICA: internal carotid artery
NIHSS: National Institute of Health Stroke Scale
PCA: posterior cerebral artery
Pcom: posterior communicating artery
PPTA: persistent primitive trigeminal artery
PT-INR: prothrombin time-international normalized ratio
SCA: superior cerebellar artery
TICI: thrombolysis in cerebral infarction
